# Results of a multi-site pragmatic hybrid type 3 cluster randomized trial comparing level of facilitation while implementing an intervention in community-dwelling disabled and older adults in a Medicaid waiver

**DOI:** 10.1186/s13012-022-01232-5

**Published:** 2022-08-26

**Authors:** Sandra L. Spoelstra, Monica Schueller, Viktoria Basso, Alla Sikorskii

**Affiliations:** 1 Kirkhof College of Nursing, Grand Valley State University, 301 Michigan St, Room C352, Grand Rapids, MI 49504 USA; 2 Statistics Department, Grand Valley State University, Grand Rapids, USA; 3 Department of Psychiatry, Michigan State University, East Lansing, USA

**Keywords:** Adoption, Sustainability, Implementation, Implementation strategies, Facilitation, Physical function, Community dwelling, Older adults, Medicaid waiver, Cluster-randomized controlled trial

## Abstract

**Background:**

Evidence-based interventions that optimize physical function for disabled and older adults living in the community who have difficulty with daily living tasks are available. However, uptake has been limited, particularly in resource-constrained (Medicaid) settings. Facilitation may be an effective implementation strategy. This study’s aim was to compare internal facilitation (IF) versus IF and external facilitation (EF) on adoption and sustainability of an intervention in a Medicaid home and community-based waiver.

**Methods:**

In a hybrid type 3 trial, waiver sites (*N* = 18) were randomly assigned to implement the intervention using a bundle of strategies with either IF or IF and EF. Adoption and sustainability were assessed via Stages of Implementation Completion (SIC) for each site. Clinician attitudes toward evidence-based practice and self-efficacy were evaluated among 539 registered nurses, social workers, and occupational therapists. Medicaid beneficiary outcomes of activities of daily living, depression, pain, falls, emergency department visits, and hospitalizations were evaluated in a sample of *N* = 7030 as reflected by electronic health records data of the Medicaid waiver program. Linear mixed-effects models were used to compare outcomes between trial arms while accounting for cluster-randomized design.

**Results:**

The mean SIC scores were 72.22 (standard deviation [SD] = 16.98) in the IF arm (9 sites) and 61.33 (*SD* = 19.29) in the IF + EF arm (9 sites). The difference was not statistically significant but corresponded to the medium clinically important effect size Cohen’s *d* = 0.60. Clinician implementation outcomes of attitudes and self-efficacy did not differ by trial arm. Beneficiary depression was reduced significantly in the IF + EF arm compared to the IF arm (*p* = .04, 95% confidence interval for the difference [0.01, 0.24]). No differences between trial arms were found for other beneficiary outcomes.

**Conclusions:**

Level of facilitation did not enhance capacity for adoption and sustainability of an evidence-based intervention in a Medicaid setting that cares for disabled and older adults. Improved beneficiary depression favored use of IF and EF compared to IF alone, and no differences were found for other outcomes. These findings also suggest level of facilitation may not have impacted beneficiary outcomes.

**Trial registration:**

ClinicalTrials.gov, NCT03634033; date registered August 16, 2018.

**Supplementary Information:**

The online version contains supplementary material available at 10.1186/s13012-022-01232-5.

Contributions to the literature

- The results inform the use of internal or internal and external facilitation as implementation strategies when adopting an evidence-based intervention in the community.
- A long-term impact may be enhancing community settings capacity for practice change, through development and testing of a bundle of implementation strategies and determining what level of facilitation is needed to enhance adoption and sustainability of evidence-based interventions.
- The methodologic approach, comparing internal facilitation versus internal and external facilitation and utilizing the Stages of Implementation Completion measure, comprised of three dimensions (quality, quantity, and timing), may be appropriate for a wide range of implementation and translational studies.

## Background

The aging population in the USA is growing rapidly [1], and nearly half report problems with physical function, which can lead to difficulty with daily tasks and often nursing home placement [2]. Evidence-based interventions that optimize physical function in settings that care for disabled and older adults are available yet rarely implemented, particularly in under-resourced settings. One such intervention is Community Aging in Place, Advancing Better Living for Elders (CAPABLE) [3–5]. CAPABLE is delivered at home by occupational therapists (OT) and registered nurses (RN) over 16 weeks using assistive devices and home modifications by a handyman to improve function and factors that impact function (e.g., balance, pain, depression). CAPABLE addresses modifiable intrinsic and extrinsic risk factors and considers the psychological, environmental, and physical factors to enhance function of disabled adults and promote aging in place [3–5].

A long-standing question in health care is how to incorporate knowledge generated from randomized controlled efficacy or effectiveness trials (like CAPABLE) into practice [6]. To close the gap, a need exists to evaluate solutions that are more effective at driving evidence into practice. Practice change and improved individual outcomes can occur with the use of proven implementation strategies [7]. Evidence on particular strategies has emerged in the literature [6]. Yet, limitations exist regarding specificity of strategies for replication. Thus, it is imperative that strategies that promote utilization of evidence be further evaluated.

One implementation strategy with demonstrated effectiveness is facilitation, a multifaceted process that involves problem-solving and supporting efforts to adopt and incorporate interventions into routine practice [8]. A facilitator acts as a “change agent” by building a supportive relationship with clinicians and providing information to enhance use of an evidence-based intervention [9, 10]. Facilitators use reflection, empathy, and counseling to deliver feedback on performance to change attitudes, skills, and behaviors of clinicians to improve practice [9, 10].

Internal facilitators are embedded in the organization or group (often a supervisor or manager), familiar with local structures, procedures, culture, and clinical processes, and perform problem-solving and support clinicians [11]. External facilitators are not a member of the organization or group, are experts in facilitation who are often linked with other facilitators, and provide guidance and support [11]. External facilitators often integrate facilitation with other implementation strategies [11]. Facilitation as both a role (a facilitator) and a process strengthens use of evidence-based interventions [12].

Several studies have shown facilitation to be an effective strategy for improving implementation of complex evidence-based practice changes and other clinical innovations, as well as improving patient and organizational level outcomes [13–16]. More specifically, trials using external facilitation (EF) have been shown to improve intervention adoption and effectiveness [14, 15]. However, a recent review of multiple trials did not delineate whether internal facilitation (IF) or EF was used [16], making it challenging to replicate findings in practice. Furthermore, mixed results on the effectiveness of facilitation are evident in the literature [17], possibly due to the intensity or dose of the facilitation strategy provided or the context in which the study occurred. Although facilitation has been widely used to address implementation challenges, limited information is available about whether IF or EF or a combination of both is more effective at adopting and sustaining practice change within a clinical setting when implementing a multimodal intervention. Hence, examining IF and EF is a topic which is of import to the field of implementation science, particularly within clinical settings with the most challenging contexts.

We conducted a hybrid type 3 cluster-randomized controlled trial that used a bundle of strategies and compared levels of facilitation while implementing an intervention. The implementation strategy bundle included relationship, coalition, and team building; readiness to implement, leadership, and clinician attitude toward evidence assessments; training; interdisciplinary coordination; audit and feedback; and IF alone or IF plus EF (IF + EF). Arm 1 included usual waiver care and used the bundle of implementation strategies that included IF alone (IF arm), while arm 2 included all components of arm 1 plus EF (IF + EF arm).

The overarching goal was to examine implementation of an intervention to improve the ability of disabled or older adults (beneficiaries) to perform daily tasks. The study had three objectives: (1) to evaluate adoption and sustainability of the intervention for two facilitation strategies, IF and IF + EF, (2) to test clinician self-efficacy and attitude toward evidence-based practice and deployment of an implementation strategy bundle on adoption and sustainability of the intervention, and (3) to examine the effect of the intervention on beneficiary outcomes. We hypothesized use of more intensive facilitation (IF + EF) would increase adoption and sustainability and improve beneficiary outcomes compared to less intensive facilitation (IF alone). Beneficiary outcomes examined were activities of daily living (ADLs), instrumental activities of daily living (IADLs), pain, depression, falls, emergency department (ED) visits, and hospitalizations.

## Methods

Study protocol is published elsewhere [18]. A brief description is as follows:

### Study design

The design was a 2-arm, 3-year pragmatic hybrid type 3 [19, 20] mixed method randomized controlled trial conducted at 18 waiver sites in the state of Michigan [21–24]. A hybrid design was chosen as it examines the effects of implementation strategies and intervention effectiveness for beneficiary outcomes [20]. The knowledge to action [24] model underpinned examination of outcomes and the Consolidated Framework for Implementation Research [25]-guided implementation.

### Study setting and participants

The settings were 18 Medicaid home and community-based waiver (HCBW) sites in Michigan that used the same electronic health record (EHR) system. Two Michigan sites using a different EHR were excluded. The waiver supports low-income (at or below 300% of the federal poverty level), nursing home eligible, disabled, and older adults in the community to avoid institutionalization. Sites care for 400 to 2500 beneficiaries and employ 10 to 125 clinicians. Sites were contracted, and clinicians were recruited via email at each site. Beneficiaries were recruited during usual care by clinicians, using a pocket aid to examine beneficiary needs in regard to the intervention. Beneficiaries could opt out and continue to receive care provided by the site, but their data were not extracted from the EHR or analyzed.

### Randomization and blinding

To assure similarity of two trial arms, sites were paired in blocks using quality assessment scores (2015–2017) and number of beneficiaries. A coin was flipped to determine arm assignment for each pair. Clinicians and beneficiaries were blinded to arm assignment.

### Power analysis

Given 18 sites (9 in each arm) available in Michigan, in the comparison of site-level outcome of adoption and sustainability, the detectable effect size with power of 0.80 in two-sided tests at .05 level of significance was Cohen’s *d* = 1.41. Given the sample size of clinicians of 539, the average cluster size was approximately 45, and with an assumed intra-class correlation coefficient (ICC) of 0.01, the design effect factor was 1.45, and the detectable effect size was Cohen’s *d* = 0.29. Given the sample size of 7030 beneficiaries, the average cluster size was approximately 390, and with an assumed intra-class correlation coefficient of 0.01, the design effect factor was 4.9, and the detectable effect size was Cohen’s *d* = 0.15 for power of 0.80 in two-sided tests at .05 level of significance. These effect sizes are below *d* = 0.33–0.5, the thresholds commonly used for clinical significance; therefore, the study was powered to detect any meaningful differences between arms on clinician and beneficiary outcomes [26, 27].

### Usual care

Usual 1915(c) HCBW services care includes annual assessments, case management, and supports coordination by RNs and social workers (SWs) via home visits and phone calls [28]. Nineteen services are provided as needed and include adult day care, chore services (e.g., cleaning, laundry), community health worker, community transportation (e.g., to doctor’s appointment), counseling, environmental modifications, and a fiscal intermediary. In addition, goods and services, home delivered meals, nursing services, personal emergency response system, private duty nursing/respiratory care, specialized medical equipment and supplies, training, personal care, medication management, lawn care, snow removal, cleaning, grocery shopping, and laundry are provided as needed.

### Intervention

In addition to the usual care that was provided, the intervention [3–5], which was previously adapted [29] to fit the Michigan HCBW, was implemented. RNs, SWs, and OTs conducted up to 10 additional home visits over 16 weeks and provided assistive devices (e.g., commode) and home modifications and alterations (e.g., installing devices or widening doorways) [29]. RNs, SWs, and OTs consulted with the individual receiving the care (beneficiary) to identify daily activity goals (e.g., taking a shower and walking to the bathroom) and evaluate barriers to achieving the goals to attain their desired outcomes, and then, care was provided. OTs assisted beneficiaries to carry out ADLs and IADLs that were challenging, such as meal preparation, bathing, and dressing. RNs targeted pain and mood management, fall prevention, incontinence prevention, and medication management. SWs addressed social and behavioral needs and issues and coordinated community resources.

### Implementation strategies

As shown in our published protocol paper [18], 9 strategies were included in the implementation bundle. A formal relationship was established by memorandum of understanding, delineating the role and duties of study staff, HCBW site, internal facilitators, and clinicians, and an informal relationship was built among those parties via monthly meetings (virtual). Readiness to implement, leadership, clinician attitude toward use of evidence, and self-efficacy were examined. Training of clinicians on use of the intervention with beneficiaries and of internal facilitators and the external facilitator on facilitation occurred. A coalition of internal facilitators met monthly (virtual) to share best practices (implementation and intervention). IF and EF are described below. Fidelity to implementation strategies was monitored, and feedback was provided to internal facilitators and the external facilitator.

#### Facilitation

Internal facilitators acted as “change agents” and “champions,” utilizing the implementation strategy bundle to support RNs, SWs, and OTs’ use of the intervention with beneficiaries (IF arm and IF + EF arm). They were identified by management teams at the sites based on the following criteria: experienced HCBW manager/supervisor (RN or SW); organized, understands the needs of others, and clear communication skills; and believed the intervention was effective. Internal facilitator training included 9-online modules on the role and tasks, problem-solving, feedback, reflection, counseling, motivational interviewing, and remediation and a 60-min session (synchronous) with the study team covering competencies [30] and the implementation plan.

Internal facilitators’ tasks (IF arm and IF + EF arm) prior to implementation included the following:

- Clarified purpose and role of internal facilitator with study staff.
- Integrated the intervention within existing clinical programs and services.
- Engaged local leadership to support implementation and use of the intervention (e.g., sign agreement).
- Reviewed the structured implementation toolkit and products (e.g., posters, emails, scripts).
- Conducted an implementation needs assessment with key stakeholders to identify potential barriers.
- Set expectations based on the local needs assessment (e.g., need to hire OT).
- Developed a localized plan (e.g., hiring OT, scheduling clinician visits) and timeline for implementation of the intervention.

Internal facilitators’ tasks (IF arm and IF + EF arm) during implementation included the following:

- Deployed the localized implementation plan (e.g., timing of training, use of OT).
- Assured RNs, SWs, and OTs completed training (online).
- Participated (virtual) in a learning collaborative (monthly) to share best practices on implementation with other internal facilitators.
- Reviewed monitoring data (weekly) on implementation status and intervention usage.
- Provided feedback to the RNs, SWs, and OTs on training and use of the intervention (weekly).
- Conducted counseling and remediation with clinicians, as needed.

The external facilitator acted as a “change agent” and was a “super-champion” at 9 HCBW sites (IF + EF arm), supporting internal facilitators to implement the intervention with clinicians. The external facilitator was an OT with 3 years of experience in facilitation and implementation of the intervention [21] in the HCBW program. External facilitator training was two-phased. First, the external facilitator completed the internal facilitator training (phase 1). Then, the external facilitator completed an online module on the external facilitator role and tasks, challenges an internal facilitator may face, and implementation barriers identified by other facilitators [31], followed by a 90-min session (synchronous) with the study team (phase 2).

During implementation, the external facilitator conducted monthly (or as needed) phone calls for up to 9 months with the 9 internal facilitators in the IF + EF arm. The external facilitator performed the following tasks with the internal facilitators:

- Mentored internal facilitators in implementation strategies, transferring the knowledge and skills necessary to support ongoing intervention sustainment.
- Reviewed the localized implementation plan and provided suggestions for uptake.
- Reviewed barriers to implementation and problem solved strategies to overcome.
- Reviewed how to apply the implementation toolkit by sharing examples used previously in the setting, so that strategies could be tailored to the local setting.
- Set expectations for use of clinicians, provided examples on differences in duties compared to usual care, and reviewed cases where the intervention was being used.
- Assisted with engagement of key stakeholders in sites to support hiring of OTs
- Monitored and provided feedback on progress in achieving implementation goals.
- Monitored use and impact of identified solutions for problems and barriers.
- Remained accessible by phone or email, as needed

It is important to note that although internal facilitators and external facilitator activities were described in a linear-phased process, the facilitation process was actually dynamic and iterative, with activities overlapping and repeating to continually monitor and adjust localized implementation processes to maximize potential for success.

### Measures

Site, clinician, and beneficiary level data were collected using the Stages of Implementation (SIC) [32, 33] (sites), Organizational readiness for change (TCU-ORC) [34], Evidence-Based Practice Attitude Scale (EBPAS) [35], general self-efficacy (GSE) [36], (clinicians), and Minimum Data Set-Home Care (MDS-HC) [37] (beneficiaries) tools as described in detail in the protocol paper [21]. We also measured clinician and beneficiary characteristics and training completion.

#### Outcomes

Primary outcome was adoption and sustainability of the intervention measured via the SIC scores. Scoring of the SIC tool (range 0–100) is described in detail in the protocol paper [18]. Secondary outcomes were clinician attitudes toward evidence-based practice and self-efficacy and beneficiary ADLs (sum of 11 ADL items), IADLs (sum of 8 IADL items), pain (sum of 4 self-reported pain items), depression (sum of 3 self-reported mood items), and number of falls, ED visits, and hospitalizations. Intervention fidelity was not collected due to difficulty extracting data from the EHR.

### Study procedures

Internal review board approvals were obtained, contracts (site, state, and EHR company) were executed, and internal facilitators (each site) and the external facilitator were selected and trained. Data (quality assessment scores, number of beneficiaries) were obtained from the state, and sites were randomized. Clinicians were recruited and consented, and baseline data (online survey; characteristics, EBPAS, GSE, TCU-ORC) were obtained. Clinicians were trained, and the intervention was provided to beneficiaries. Clinician EBPAS and GSE were collected again at 9 months after completion of training (June 30, 2020). We planned to collect the SIC (phone surveys) data monthly for 12 months; however, due to COVID, data were not collected in April through August 2020, with the exception of 3 surveys from some of the sites. Beneficiary data were extracted from the EHR, which included the last assessment prior to the clinician training (before October 1, 2019) and assessments following clinician training (October 2, 2019, to June 30, 2020).

### Data analysis

Stages of implementation scores were compared between trial arms using *t*-tests, and effect sizes (Cohen’s d) were estimated as differences between means expressed in standard deviation units. The cutoffs for the interpretation of Cohen’s d are 0.2 (small), 0.5 (medium), and 0.8 (large) [38]. Characteristics of clinicians and beneficiaries were summarized by trial arm at baseline. Because of the turnover of clinicians at each site, constrained longitudinal model, with measures at baseline and at 9 months and a constraint of equality of means at baseline due to randomization, was used for the analysis of clinician data. With this analytical technique, data from all clinicians who completed baseline only, 9 months only, or both surveys were used. Random effects were used to account for nesting of clinicians within sites. For all beneficiaries, baseline data were available, and characteristics of beneficiaries without post-intervention data were compared by trial arm to evaluate potential bias due to missing values. Post-intervention data were analyzed in relation to trial arm with the adjustment for baseline version of the outcome, age, sex, and any baseline factors that differed in terms of missing values.

## Results

### Site adoption and sustainability (objective 1)

All waiver sites (*N* = 18; 100%) remained engaged in the trial during the entire study, with a mean of 418.3 days (standard deviation [SD] 11.54; range 224–496). The mean SIC scores (range 0–100) were 72.22 (*SD* = 16.98) in the IF arm and 61.33 (*SD* = 19.29) in the IF + EF arm. The difference was not statistically significant (*p* = 0.22) with the sample size of 9 per arm but corresponded to the medium clinically important effect size Cohen’s *d* = 0.60.

### Clinician implementation outcomes (objective 2)

**Fig. 1:**
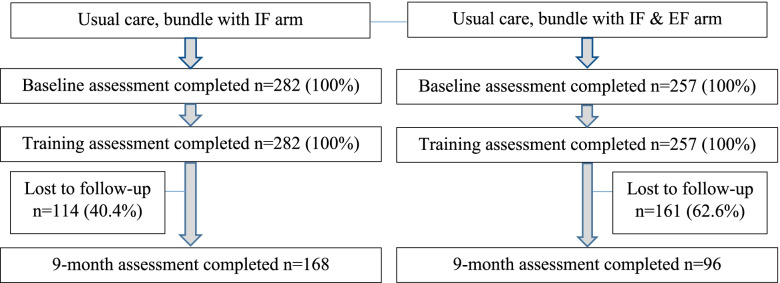
Consort diagram of clinician study participants

There were 539 clinicians (*n* = 282 IF arm, *n* = 257 IF + EF arm) that comprised the study sample at baseline (see Fig. 1). As shown in Tables 1 and 2, clinicians had similar sociodemographic characteristics and outcome values at baseline in two trial arms. All 539 (100%) of the clinicians completed baseline and knowledge uptake surveys at baseline. Two-hundred and sixty-four clinicians completed the 9-month survey (*n* = 168 in the IF arm; *n* = 96 in the IF + EF arm). A total of 312 clinicians (*n* = 133 in the IF arm, *n* = 179 in the IF + EF arm) had baseline data but not the 9-month measure, whereas 37 clinicians (*n* = 19 in the IF arm, *n* = 18 in the IF + EF arm) had the 9-month measure but not baseline data. The sample ICCs at baseline were below the value of .01 planned in a priori power analysis and were equal to .005 for self-efficacy (GSE) and .0007 for attitudes (EPBAS). There were no differences in clinician outcomes at month 9 (Table 3).

**Table 1:** Sociodemographic characteristics of the clinician sample (*n* = 539)

	***IF, n*** **(%)**	***IF + EF, n (%)***	***All, n (%)***
**Race**			
American Indian or Alaskan Native	5 (1)	1 (< 1)	6 (1)
Asian	1 (< 1)	4 (1)	5 (1)
Black or African American	14 (3)	39 (7)	53 (10)
White	248 (46)	202 (37)	450 (83)
More than one race	10 (2)	4 (1)	14 (3)
**Ethnicity**			
Hispanic	13 (2)	5 (1)	18 (3)
Non-Hispanic	267 (50)	247 (46)	514 (95)
**Discipline**			
Registered nurse (RN)	137 (25)	111 (21)	248 (46)
Social worker (SW)	137 (25)	132 (24)	269 (50)
Occupational therapist (OT)	2 (< 1)	4 (1)	6 (1)
**Gender**			
Male	16 (3)	22 (4)	38 (7)
Female	266 (49)	231 (43)	497 (92)
**Highest degree**			
Associates	86 (16)	53 (10)	139 (26)
Bachelors	133 (25)	101 (19)	234 (43)
Masters	57 (11)	92 (17)	149 (28)
**Years worked in healthcare**			
< 1	3 (1)	5 (1)	8 (1)
1–5	64 (12)	37 (7)	101 (19)
6–10	55 (10)	43 (8)	98 (18)
11–15	31 (6)	33 (6)	64 (12)
16–20	36 (7)	33 (6)	69 (13)
21–25	39 (7)	37 (7)	76 (14)
26–30	28 (5)	23 (4)	51 (9)
> 30	25 (5)	45 (8)	70 (13)
**Years worked in waiver**			
< 1	44 (8)	28 (5)	72 (13)
1–5	148 (27)	122 (23)	270 (50)
6–10	50 (9)	45 (8)	95 (18)
11–15	13 (2)	19 (4)	32 (6)
16–20	11 (2)	22 (4)	33 (6)
21–25	9 (2)	17 (3)	26 (5)
26–30	4 (1)	2 (< 1)	6 (1)
	**Mean (SD)**		
Age	43.07 (11.57)	47.00 (11.59)	44.92 (11.74)

**Table 2:** Baseline values of the outcomes and potential mediators by trial arm for clinicians

***Outcome or mediator***	***IF mean (SD)***	***IF + EF mean (SD)***
Clinician climate	35.78 (6.06)	35.02 (6.61)
Clinician culture	12.54 (1.92)	12.35 (2.19)
Clinician training	8.18 (1.61)	8.12 (1.56)
Clinician motivation	13.25 (3.40)	13.95 (3.28)
Clinician pressure to change	10.49 (2.30)	9.70 (2.48)
Clinician leadership	34.11 (9.07)	32.91 (10.21)
Clinician attitude	105.90 (18.27)	106.25 (19.10)
Clinician self-efficacy	32.20 (3.80)	32.19 (3.84)
Beneficiary ADL	32.05 (16.76)	33.48 (15.90)
Beneficiary IADL	37.95 (10.04)	37.28 (9.41)
Beneficiary pain	2.56 (3.08)	3.01 (3.20)
Beneficiary depression	1.44 (1.99)	1.05 (1.79)
	***n*** **(%)**	***n*** **(%)**
Beneficiary falls	177 (22)	113 (13)
Beneficiary ED visits	450 (13)	421 (12)
Beneficiary hospitalizations	495 (14)	450 (13)

**Table 3:** Post-intervention differences between trial arms for clinicians

***Outcome or mediator***	***IF mean change (SE)***	***IF + EF mean change (SE)***	***Difference between arms (95% CI)***	***p-Value differences between arms***
Clinician self-efficacy	−2.34 (0.21)	−2.57 (0.30)	0.23 (−0.51, 0.97)	0.54
Clinician attitude — fit	*	*	*	*
Clinician attitude — limitations	0.00 (0.11)	−0.06 (0.11)	0.06 (−0.27, 0.39)	0.68
Clinician attitude — openness	0.08 (0.08)	0.00 (0.08)	0.08 (−0.13, 0.29)	0.45
Clinician attitude — monitoring	0.01 (0.14)	0.18 (0.14)	−0.17 (−0.66, 0.32)	0.44
Clinician attitude — requirements	0.15 (0.09)	0.09 (0.10)	0.06 (−0.22, 0.35)	0.64
Clinician attitude — employability	−0.17 (0.07)	−0.04 (0.11)	−0.12 (−0.36, 0.11)	0.29
Clinician attitude — feedback	0.10 (0.11)	−0.01 (0.12)	0.11 (−0.24, 0.47)	0.50
Clinician attitude — burden	0.13 (0.15)	0.04 (0.14)	0.09 (−0.42, 0.59)	0.69
Clinician attitude — appeal	0.06 (0.08)	−0.11 (0.08)	0.17 (−0.06, 0.41)	0.13
Clinician attitude — divergence	0.07 (0.09)	0.14 (0.09)	−0.07 (−0.31, 0.17)	0.57
Clinician attitude — balance	0.11 (0.11)	0.21 (0.10)	−0.10 (−0.47, 0.27)	0.54
Clinician attitude — total	−103.49 (0.92)	−103.52 (0.92)	0.03 (−0.08, 0.14)	0.70

^*^Algorithm did not converge

### Beneficiary outcomes (objective 3)

**Fig. 2:**
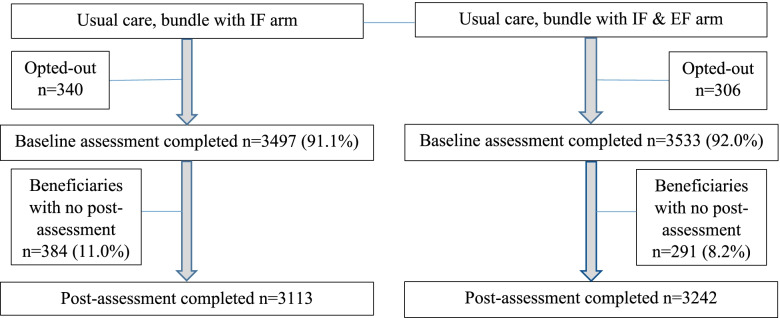
Consort diagram of beneficiary study participants

The sample of 7030 beneficiaries (*n* = 3497 IF arm; *n* = 3533 IF + EF arm) represented 9752 beneficiaries served by the participating sites as of May 15, 2019. Of the beneficiaries served, 7676 had the assessment near baseline of the trial (before October 1, 2019), 646 opted out (*n* = 340 IF arm; *n* = 306 IF + EF arm, Fig. 2), and the characteristics of 7030 are in Table 4. Of these 7030 beneficiaries, 384 in the IF arm and 291 in the IF + EF arm did not have the second (post-intervention) MDS-HC assessment. The sample ICCs ranged from .01 (pain) to .039 (IADL) across beneficiary outcomes. The only significant difference by trial arm among beneficiaries with no post-intervention assessment was on age (*p* = .04, Supplemental Table 1). Despite cluster randomization, IF + EF arm had a sizably larger proportion of African-American participants (33% versus 15% in the IF arm, Table 4), lower rate of recent falls (13% versus 22%, Table 2), and lower pain intensity at baseline (Table 2). Per CONSORT guidelines, significance testing at baseline was not performed, but baseline recent falls and pain intensity were controlled for along with age and sex in the linear mixed-effects models for post-intervention outcomes. Among the post-intervention outcomes, only depression was significantly lower in the IF + EF group compared to IF (*p* = .04, Table 5). No harms or unintended negative events attributed to the intervention were reported by beneficiaries.

**Table 4:** Sociodemographic characteristics of the beneficiary sample (*n* = 7030)

	***IF, n*** **(%)**	***IF + EF, n (%)***	***All, n (%)***
**Race**			
American Indian or Alaskan Native	25 (< 1)	9 (< 1)	34 (1)
Asian	25 (< 1)	25 (< 1)	50 (1)
Black or African American	541 (8)	1228 (17)	1769 (25)
Hawaiian or South Pacific	3 (< 1)	4 (< 1)	7 (< 1)
White	2735 (39)	2122 (30)	4857 (69)
More than one race	49 (1)	33 (1)	82 (1)
**Ethnicity**			
Hispanic	108 (2)	97 (1)	205 (3)
Non-Hispanic	3357 (48)	3401 (48)	6758 (96)
**Gender**			
Male	1137 (16)	1130 (16)	2267 (32)
Female	2360 (34)	2403 (34)	4763 (68)
	**Mean (SD)**		
Age	69.50 (14.58)	71.19 (14.02)	70.35 (14.32)

**Table 5:** Post-intervention differences between trial arms for beneficiaries

***Outcome***	***IF LS mean (SE)***	***IF + EF LS mean (SE)***	***Difference between arms (95% CI)***	***p-value for differences between arms***
ADL	26.13 (0.20)	25.85 (0.19)	(−0.16, 0.73)	0.73
IADL	29.54 (0.17)	29.24 (0.17)	(−0.11, 0.72)	0.15
Pain	7.52 (0.13)	7.44 (0.13)	(−0.23, 0.39)	0.61
Pain intensity	2.71 (0.08)	2.70 (0.08)	(−0.18, 0.20)	0.92
Depression	1.30 (0.05)	1.17 (0.05)	(0.01, 0.24)	0.04
	**Adjusted rate (SE)**	**Adjusted rate (SE)**	**OR (95%** ***CI*) for IF vs. IF + EF (1 vs. 2)**	***p*-value for differences between arms**
Falls	15.44%	13.56%	1.16 (0.93, 1.45)	0.18
Recent falls	5.40%	4.62%	1.18 (0.71, 1.95)	0.52
ED visits	0.92%	0.90%	0.98 (0.79, 1.22)	0.86
Hospitalizations	1.48%	1.44%	1.01 (0.72, 1.42)	0.95

## Discussion

This trial sets an ambitious agenda to explore ways to adopt and sustain an evidence-based intervention across a Medicaid program. There was a significant amount of work that presented challenges and opportunities. First, implementation and evaluation with a hybrid 3 design in an under-resourced real-world setting forced a delicate balance of study design and voluntary participation. Our work with sites in a standardized manner encouraged local tailoring of the plan to optimize implementation. Similar to other studies, this trial was a success from a design perspective in that no sites dropped out [39]. Second, the selection of volunteer sites may limit heterogeneity in our sample. However, findings about stages of implementation will inform this intervention’s adoption and sustainability and may be generalizable to other settings or populations. Furthermore, findings from this trial are likely to generalize to other states and some community settings implementing the intervention because many challenges may be similar to our low-resource Medicaid setting. Third, this trial used 9 evidence-based implementation strategies; finding use of IF alone has less up-front cost and appears more sustainable. Like other trials, multiple strategies were needed to achieve adoption and sustainability [40, 41], and level of facilitation may or may not have impacted implementation [10, 14–17]. Fourth, the measurement approach involved significant effort in data collection from sites, clinicians, and beneficiaries with surveys, interviews, and extraction of secondary administrative and clinical data across disparate clinician contexts. Despite the challenges of data collection and management, the findings generate a broader understanding in Medicaid settings and facilitate effective use of evidence in under-resourced, complex environments. Finally, past trials of the efficacy of CAPABLE with respect to beneficiary outcomes had similar results [3–5, 42]. We expected to learn a great deal about beneficiary outcomes to further support efficacy of CAPABLE but were unable to collect intervention fidelity data due to difficulty extracting data from the EHR.

Similar to other studies, we believe that this methodological approach of measuring adoption and sustainability with the SIC, which is comprised of the three dimensions of quality, quantity, and timing, is appropriate for a wide range of implementation and translational studies [39]. We note that the Michigan Department of Health and Human Services Long-Term Care Division played a critical role in use of the intervention and implementation. Other states or settings may not have access to such an organization to support implementation.

### Limitations

The trial was limited by the number of sites in the state that utilized the same EHR (18 of 20). For this reason, only large effect sizes for site-level outcome of adoption and sustainability were detectable. To address this limitation, we also estimated the effect size to inform future dissemination and implementation trials. The ICCs for beneficiaries nested within sites were higher than planned based on the literature for outcomes other than pain. The nesting of beneficiaries within sites was accounted for analytically, but higher ICCs resulted in larger detectable effect sizes than planned in the a priori power analysis. Despite this limitation, observed point estimates of the differences between trial arms were small in magnitude, including the one significant difference between trial arms on beneficiary depression. Therefore, meaningful conclusions from this trial were not affected by somewhat higher than anticipated ICCs. Like other studies, we relied on self-reported data to measure clinician and beneficiary constructs [43, 44]. We took several steps to mitigate this bias, including reminding the clinicians that their responses would not be shared with others and using standardized data collection tools. In addition, the last few months of the trial may have been impacted by COVID, as IF and EF were not as intense as planned due to competing demands and short staffing at sites [45], yet the overall design and intervention implementation were sustained. Finally, even though all outcomes for clinicians and beneficiaries were specified a priori, one significant finding on beneficiary depression may be best explained by chance, supporting the overall conclusion of no differences between levels of facilitation.

## Conclusions

In what may be the first randomized controlled trial examining implementation of the evidence-based CAPABLE intervention, the level of facilitation did not enhance the capacity for adoption and sustainability in an under-resourced Medicaid setting that cares for disabled and older adults. This may suggest that only internal facilitation may be warranted. These findings further support the merits of less intensive implementation approaches that ensure adequate training and ongoing facilitation to support clinicians attempting to implement a new intervention. Future studies should evaluate factors that predict optimal implementation, particularly internal facilitation.

## Supplementary Information

**Additional file 1: Supplemental Table 1.** Baseline characteristics of beneficiaries with no post-intervention assessment by trial arm.

## Data Availability

The datasets generated during and/or analyzed will be available upon request from the investigators.

## Abbreviations

ADL: Activities of daily living
EHR: Electronic health record
ED: Emergency department
EBPAS: Evidence-Based Practice Attitude Scale
EF: External facilitation
GSE: General self-efficacy
IADL: Instrumental activities of daily living
IF: Internal facilitation
MDS-HC: Minimum Data Set-Home Care
OT: Occupational therapist
TCU-ORC: Organizational readiness for change
PI: Principal investigator
RN: Registered nurse
SW: Social worker
SIC: Stages of implementation completion
