# Supplementary material for: Results of a multi-site pragmatic hybrid type 3 cluster randomized trial comparing level of facilitation while implementing an intervention in community-dwelling disabled and older adults in a Medicaid waiver
Source: Implement Sci. 2022 Aug 26;17:57. doi: 10.1186/s13012-022-01232-5 (PMC9419328; doi:10.1186/s13012-022-01232-5)
Supplement: Supplementary file 1 — Additional file 1: Supplemental Table 1. Baseline characteristics of beneficiaries with no post-intervention assessment by trial arm. [file 13012_2022_1232_MOESM1_ESM.docx]

**SUPPLEMENTARY FILE**

Supplemental Table 1

| ***Outcome*** | ***IF Mean (SE) or n (%)*** | ***IF+EF Mean (SE) or n (%)*** | ***p-value for differences between arms*** |
| --- | --- | --- | --- |
| **Race** |  |  |  |
| American Indian or Alaskan Native | 4 (1) | 1 (<1) | 0.31 |
| Asian | 4 (1) | 2 (1) | 0.49 |
| Black or African American | 57 (15) | 77 (26) | 0.35 |
| Hawaiian or South Pacific | 1 (<1) | 1 (<1) | 0.84 |
| White | 304 (79) | 190 (65) | 0.49 |
| More than one race | 4 (1) | 2 (1) | 0.63 |
| Did not respond | 10 (2) | 18 (6) |  |
| **Ethnicity** |  |  | 0.72 |
| Hispanic | 10 (3) | 8 (3) |  |
| Non-Hispanic | 369 (96) | 283 (97) |  |
| Missing | 5(1) | 0 (0) |  |
| **Gender** |  |  | 0.12 |
| Male | 141 (37) | 86 (30) |  |
| Female | 243 (63) | 205 (70) |  |
|  | **Mean (SD)** | **Mean (SD)** |  |
| Age | 67.46 (15.76) | 70.64 (15.17) | 0.04 |
|  |  |  |  |
| ADL | 23.70 (16.07) | 24.48 (14.63) | 0.56 |
| IADL | 29.04 (8.84) | 29.30 (8.51) | 0.51 |
| Pain | 7.56 (6.00) | 7.78 (5.86) | 0.51 |
| Pain intensity | 2.36 (2.98) | 2.69 (3.15) | 0.23 |
| Depression | 1.51 (2.12) | 1.04 (1.79) | 0.20 |
|  | **n (%)** | **n (%)** |  |
| Falls | 92 (24) | 51 (18) | 0.07 |
| Recent falls | 29 (21) | 10 (17) | 0.47 |
| ED visits | 52 (14) | 31 (11) | 0.99 |
| Hospitalizations | 66 (17) | 33 (11) | 0.10 |
